# The Need for Better Statistics in *Animals*. Comment on Phillips, C.J.C. Quality, Integrity, and Transparency in Animal Science: The *Animals* Perspective. *Animals* 2026, *16*, 403

**DOI:** 10.3390/ani16132082

**Published:** 2026-07-06

**Authors:** Richard A. Laven

**Affiliations:** School of Veterinary Science, Massey University, Palmerston North 42111, New Zealand; r.laven@massey.ac.nz

Clive Philips’s commitment to making *Animals* a high-quality journal is absolutely fundamental to the success of the journal, and undoubtedly one of the reasons that it has a high impact despite publishing a very large number of papers.

As such, his Editorial on quality, transparency, and integrity in animal science [1] is a very welcome statement of the principles underlying the quality of the papers published in *Animals*. However, in one section, “Statistical Analysis: Clarity over Complexity”, the outlined recommendations do not match current best practice for the interpretation of statistical outputs.

The first paragraph’s call for clarity over complexity is clear and well-argued. If statistics are complex, then they need to be explained so that the reader can be reassured that the results are valid and meaningful. Unfortunately, the second paragraph then presents a standard misinterpretation of *p*-values. Firstly, the claim is made that if *p* > 0.05, then results should not be presented as definitive. However, this artificial dichotomisation implies “a false distinction between outcomes and creates the drive to obtain “significant” results regardless of objective reality” [2,3]. A study with a *p*-value of 0.051 is fundamentally no different from one with a *p*-value of 0.049. Furthermore, just because a result is “significant” at *p* < 0.05, does not meant that it is actually “significant” (i.e., important). It is also important to note that a non-significant *p*-value can occur because the study lacked the power to detect any meaningful difference (so the only conclusion is “we do not know”, not “there was no association”) or because the study demonstrated that biologically important effects were unlikely. Reporting effect size (e.g., mean difference or odds ratios) and confidence intervals (CIs) can solve those problems as all the “values in a conventional 95% interval can be described as highly compatible with data under the background statistical assumptions, in the very narrow sense of having *p* > 0.05 under those assumptions” [4]. We need to move away from treating *p* < 0.05 as a meaningful threshold as “[o]vercoming the binary logic of *p*  <  0.05 is essential to enhance transparency [and] reduce bias” (Ferreira et al. 2026) [5].

I fully appreciate that reducing the use of the *p*-value as a means of dichotomising results into “significant” and “non-significant” is a challenge, as it has been ingrained into most authors who publish in *Animals* (who are not statisticians) as the approach to use. The second error in the statistical analysis section is much easier to prevent. This is the claim that *p*-values between 0.05 and 0.1 indicate a trend towards significance, most commonly expressed in the rote statement “The threshold for significance was set at *p* ≤ 0.05, and trends were reported at *p* < 0.10”. This is both an empty exercise in semantics [6] and an error that is “neither trivial nor merely semantic” [7].

A single outcome has one *p*-value, whereas a “trend” (or the related “tendency”) needs multiple results pointing in one direction. “Trend” is most commonly used to suggest that if more results were available, the study would have become significant. This is untrue. We can calculate the probability that increasing numbers lower *p*-values, as this probability is dependent on the *p*-value of the original study and the relative amount of additional data [8]. For a *p*-value of 0.08, if we added 20% more data, our subsequent second *p*-value would be >0.05 54% of the time. Even with 100% more data, it would still be >0.05 30% of the time. I still remember the comment of an author in response to this 30% figure, saying that they had a 70% chance of being right. This is both a misunderstanding of statistics—the 70% refers to the probability averaged across an infinite number of studies, where each individual study will have a *p*-value either above or below 0.05 once the data are added—and the acceptance of a 70% threshold rather than the 95% one they were conditioned to use. Furthermore, the outcome that they are focusing on, *p* < 0.05, is still an arbitrary threshold that does not necessarily indicate that the difference would be a meaningful one.

Banning the use of the term “trend” to mean anything other than multiple results is simple and easy to achieve. It is also a good first step in getting scientists to understand that dichotomisation by *p*-value is a poor practice. Therefore, it is disappointing to see it mentioned by Clive Philips as a suitable approach in his Editorial.

Statistical misinterpretation is not a problem confined to *Animals*. During a recent review of the topic [9], we identified that statistical misinterpretation is common across many animal science journals. However, my personal experience of reviewing and editing for *Animals* is that the standard of statistics in papers submitted is much poorer than in the other high-impact journals that I have reviewed and edited for. I also find that when I am the reviewer, comments on statistics are more likely to be ignored by editors than such comments in other journals. As such, it is additionally disappointing that the Editorial promulgated statistical inference that is not best practice.
